# Editorial: Overcoming genome editing challenges in plants: new tools and nanotechnologies

**DOI:** 10.3389/fgeed.2023.1230424

**Published:** 2023-06-01

**Authors:** Sachin Rustgi, Huan Zhang, Tufan Mehmet Oz

**Affiliations:** ^1^ Department Plant and Environmental Sciences, School of Health Research, Clemson University Pee Dee Research and Education Center, Florence, SC, United States; ^2^ School of Agriculture and Biology, Shanghai Jiao Tong University, Shanghai, China; ^3^ Earlham Institute, Norwich Research Park, Norwich, Norfolk, United Kingdom

**Keywords:** genome editing, nanotechnology, biomolecule delivery systems, molecular diagnostic, CRISPR

Genome editing has been an active research area for the last 2 decades (Carroll, 2021). As a result, we have witnessed many breakthroughs, from the development of designer nucleases to their use in microbes, animals, humans, and agricultural plants (Adli, 2018; Zhang et al., 2019; Anzalone et al., 2020; Li et al., 2023; Wang and Doudna, 2023). More recently, to improve the editing accuracy and precision and reduce dependence on the cell’s developmental state, new approaches, such as the OMEGA (obligate mobile element-guided activity), CAST (CRISPR-Cas-associated transposon), and INTEGRATE (insertion of transposable elements by guide RNA-assisted targeting), were developed and tested in different organisms (Tenjo-Castaño et al., 2022). Further, CRISPR technology was deployed in imaging, diagnostics (Wang and Doudna, 2023), and treatment of major human disorders (Wang and Doudna, 2023). Likewise, these technologies were deployed in agriculture to edit all major row crops, such as rice, wheat, maize, cotton, soybean, and horticultural crops like banana, tomato, apple, and poplar, for various traits from disease resistance to consumer preference traits (Zhu et al., 2020; FAO, 2023).

Despite the revolutionary nature of genome-editing tools and the notable progress that these tools have enabled in plant improvement, there remain many challenges for the mainstream application of CRISPR technology in many plant species. Most of these challenges stem directly or indirectly from the cargo delivery and tissue culture-based plant regeneration bottlenecks (Rustgi et al.). Recent progress has been made in the delivery area through using nanomaterials and DNA/RNA viruses, along with notable improvements to the tissue culture process via developmental regulators, growth factors, and haploidy inducers, among many other approaches (Rustgi et al.).

Given the remarkable amount of research on genome editing, there are still some bottlenecks, making it imperative to summarize the progress and identify areas that need further research. Keeping this goal in mind, we invited research and review papers from the leading research group in this workspace. After the extensive peer-review process, five articles that summarize the depth of the subject area were published. The articles published in this volume are briefly summarized below.

In this volume, the article by Rustgi et al. provides a detailed insight into different biomolecule delivery methods. The authors have classified biomolecule delivery into conventional biomacromolecule delivery, unconventional gene delivery, nanoparticle-based gene delivery, cell-penetrating peptides, virus-mediated delivery, direct delivery, and pollen/microspore-mediated gene delivery methods. The conventional delivery methods and different alterations of these methods proved advantageous in specific situations. The authors also discussed using the electric field, magnetic field, sonication, silicon carbide whiskers, fungi, and bacteria for biomolecule delivery under unconventional methods.

A great deal of information about nanoparticle-based gene delivery methods was summarized in the article. It deals with delivering DNA, RNA, and protein cargoes into cells in leaves and pollens by carbon nanotubes, DNA nanostructures, or gold nanoparticles. The paper also discussed the topical delivery of siRNA and dsRNA via coating them onto BioClay or carbon nanodots. The paper summarizes the pros and cons of these delivery methods.

The use of viruses for the delivery of genome editing reagents is commonplace in animal and human cells, but it is slowly gaining popularity in plants. The authors discussed the various available plant viruses, their cargo capacities, host range, and their application in genome editing and transient gene silencing for gene function characterization.

Under the direct delivery methods, authors discuss the delivery of genome editing reagents as ribonucleoprotein complex and direct delivery of double-stranded RNA and Fluoroarabino Nucleic Acid Antisense Oligonucleotides (FANA-ASOs) to target essential pest and pathogen genes to plants to manage these pests and pathogens. At the end of the article, the authors talked about plant-specific pollen-based methods for delivering genome editing reagents.

In sum, this article provided a broad overview of the available biomolecule (DNA, RNA, protein, and ribonucleoprotein complex) delivery methods to the readers and discussed the pros and cons of different methods allowing the readers to make the informed choice of the method for their specific experiments and talked about prospects and identified potential areas where more research is needed.

Lately, the field of plant nanobiotechnology has advanced significantly in the design of nanoparticles for biomolecule cargo delivery and genetic engineering (Demirer et al., 2021; Savage, 2022). Meanwhile, the characterization of how nanoparticles interact with and enter plant cells remained an area of great interest and drove research in this enterprise. Two papers in the current volume, by Sharma and Lew and Zhao et al. explored these advancements and their implications for plant research.

In the first paper, Sharma and Lew highlighted the importance of designing nanoparticles with specific properties that can help unlock the full potential of CRISPR/Cas technology in targeted manipulation of the plant genome to improve agricultural output. The authors discussed how nanoparticles could be engineered to improve the delivery of nucleic acids to plant cells.

The implications of this research are significant, as the ability to efficiently deliver genome editing components to plant cells can greatly accelerate the development of new crop varieties with improved traits. Additionally, using biodegradable materials can alleviate concerns about the potential adverse effects of nanoparticles on the environment. However, we should pay attention to the implications of the regulatory frameworks and social acceptance of nano-enabled precision breeding in the future.

In the second paper, Zhao et al. explored the development of imaging tools that enable researchers to study plant nanoparticle interactions at three-dimensional lengths from micro-to-macro scale, including nanoparticle movement within plant organs, tissues, cells, and subcellular organelles. The authors discussed various imaging techniques, and by comparing the benefits and limitations of different optical systems, the authors proposed promising imaging tools for plant nanobiotechnology and their applications in plant research.

The development of imaging tools for plant nanobiotechnology has opened up new avenues for understanding how nanoparticles interact with plant cells and tissues. Meanwhile, different imaging techniques can detect and quantify nanoparticle absorption, transportation, and accumulation in plants with different penetration abilities, penetration timing, and spatial resolution. This information is critical for designing nanoparticles that can effectively deliver genome editing components to the desired location within the plant. Additionally, these imaging tools can be used to study the potential effects of nanoparticle use on plant growth and development.

Overall, these two papers demonstrate the importance of nanotechnology in plant biology. The design of nanoparticles with specific properties coupled with the development of imaging tools has the potential to revolutionize the field of crop improvement. However, one thing that needs to be considered is nanoparticle’s short- or long-term effects on plants. By combining multiple approaches, researchers can better understand how to design both effective and safe nanoparticles for plant research.

As mentioned earlier, some challenges for the widespread application of genome editing in plants are associated with cargo delivery, plant regeneration, or the introduction of intended mutations in cell types that result in heritable germline mutations. Researchers have developed tools such as engineered viral vectors to address some of these challenges. The manuscript by Beernink et al. hypothesized that RNA mobility signals facilitate the entry of engineered viruses carrying gene editing cargo into the shoot apical meristem, where germline mutations can occur. Engineered foxtail mosaic virus (FoMV) was successfully used earlier to deliver functional sgRNAs in plants. However, the ability of RNA mobility sequences, such as Flowering Locus T (FT) and tRNA, to promote FoMV-induced somatic and germline mutations remains to be explored. In their report, Beernink et al. summarized their work investigating the impact of RNA mobility signals on virus-induced germline gene editing in *Nicotiana benthamiana* and *Zea mays*. Altogether, their results indicated that RNA mobility signal, such as FT, fused to sgRNA was insufficient to facilitate virus-induced germline mutations. Therefore, a detailed investigation of compatible virus-host combinations and analysis of mobility, editing, and heritability mechanisms are critical to achieving virus-induced germline gene editing.

Another promising approach to address challenges related to gene editing and heritability in plants relies on the choice of the promoter used to drive gRNA and Cas9 expression. It is hypothesized by Rahman et al. that spatiotemporal regulation of Cas9 expression using tissue-specific or inducible promoters enables higher heritability and efficiency of targeted mutagenesis with reduced off-target effects. In their review, Rahman et al. concluded that spatiotemporal regulation of Cas9 enabled greater accuracy and heritability than constitutive promoters. They also pointed out that most studies were conducted in *Arabidopsis* but not crop species which mainly depend on tissue culture procedures for transgenic or edited event generation. Therefore, a comparative investigation of spatiotemporally-regulated promoters across different plant species must assess the broader applicability of tissue-specific and inducible promoters beyond *Arabidopsis*.
